# Real-World Safety and Outcome of First-Line Pembrolizumab Monotherapy for Metastatic NSCLC with PDL-1 Expression ≥ 50%: A National Italian Multicentric Cohort (“*PEMBROREAL*” Study)

**DOI:** 10.3390/cancers16101802

**Published:** 2024-05-08

**Authors:** Alessandro Cafaro, Flavia Foca, Oriana Nanni, Marco Chiumente, Marina Coppola, Alberto Russi, Elena Svegliati, Paolo Baldo, Sabrina Orzetti, Fiorenza Enrico, Federico Foglio, Davide Pinnavaia, Vito Ladisa, Claudia Lauria Pantano, Rosa Lerose, Patrizia Nardulli, Simona Ferraiuolo, Piera Maiolino, Immacolata De Stasio, Federica Gradellini, Anna Rita Gasbarro, Rossella Santeramo, Gisella Carrucciu, Riccardo Provasi, Mario Cirino, Paola Cristina Cappelletto, Elisabetta Fonzi, Alessandra Pasqualini, Stefano Vecchia, Marianna Veraldi, Adele Emanuela De Francesco, Lucio Crinò, Angelo Delmonte, Carla Masini

**Affiliations:** 1 Pharmacy Unit, IRCCS Istituto Romagnolo per lo Studio dei Tumori (IRST) “Dino Amadori”, 47014 Meldola, Italy; carla.masini@irst.emr.it; 2 Unit of Biostatistics and Clinical Trials, IRCCS Istituto Romagnolo per lo Studio dei Tumori (IRST) “Dino Amadori”, 47014 Meldola, Italy; flavia.foca@irst.emr.it (F.F.); oriana.nanni@irst.emr.it (O.N.); 3 Scientific Direction, Società Italiana di Farmacia Clinica e Terapia (SIFaCT), 10123 Turin, Italy; marco.chiumente@sifact.it; 4 Pharmacy Unit, IRCCS Istituto Oncologico Veneto (IOV), 35128 Padova, Italy; marina.coppola@iov.veneto.it (M.C.); alberto.russi@iov.veneto.it (A.R.); elena.svegliati@gmail.com (E.S.); 5 Pharmacy Unit, CRO Aviano IRCCS, National Cancer Institute, 33081 Aviano, Italy; pbaldo@cro.it (P.B.); sabrina.orzetti@cro.it (S.O.); 6 Hospital Pharmacy, Candiolo Cancer Institute, FPO-IRCCS, Candiolo, 10060 Turin, Italy; fiorenza.enrico@ircc.it (F.E.); federico.foglio@ircc.it (F.F.); davide.pinnavaia@ircc.it (D.P.); 7 Hospital Pharmacy, IRCCS National Cancer Institute Foundation, 20133 Milan, Italy; vito.ladisa@istitutotumori.mi.it (V.L.); claudia.lauria@istitutotumori.mi.it (C.L.P.); 8 Hospital Pharmacy, IRCCS-CROB Referral Cancer Center of Basilicata, 85028 Rionero in Vulture, Italy; rosa.lerose@crob.it; 9 Pharmacy Unit, National Cancer Research Center Istituto Tumori “Giovanni Paolo II”, 70121 Bari, Italy; p.nardulli@oncologico.bari.it (P.N.); s.ferraiuolo@oncologico.bari.it (S.F.); 10 Pharmacy Unit, Istituto Nazionale Tumori “Fondazione G. Pascale”, IRCCS, 80131 Naples, Italy; p.maiolino@istitutotumori.na.it (P.M.); i.destasio@istitutotumori.na.it (I.D.S.); 11 Pharmacy Unit, Azienda USL-IRCCS di Reggio Emilia, 42122 Reggio Emilia, Italy; federica.gradellini@ausl.re.it; 12 Pharmacy Unit, University Hospital Policlinico, 70100 Bari, Italy; gasbarroannarita17@gmail.com (A.R.G.); rossella.santeramo@policlinico.ba.it (R.S.); 13 Pharmacy Unit, Azienda Ospedaliera Brotzu, 09047 Cagliari, Italy; gisella.carrucciu@gmail.com; 14 Department of Pharmacy, Institute for Maternal and Child Health-IRCCS “Burlo Garofolo”, 34137 Trieste, Italy; riccardo.provasi@gmail.com (R.P.); mario.cirino@asugi.sanita.fvg.it (M.C.); 15 Hospital Pharmacy, Hospital of Bolzano (SABES-ASDAA), 39100 Bolzano-Bozen, Italy; paolacristina.cappelletto@sabes.it; 16 Pharmacy Unit, S.Chiara Hospital, 38122 Trento, Italy; elisabetta.fonzi@apss.tn.it (E.F.); alessandra.pasqualini@apss.tn.it (A.P.); 17 Pharmacy Unit, Hospital Guglielmo da Saliceto, 29121 Piacenza, Italy; s.vecchia@ausl.pc.it; 18 Protesic and Pharmaceutical Assistance Sector n. 3, Department of Health Protection and Health Service Calabria Region, 88100 Catanzaro, Italy; marianna.veraldi@regione.calabria.it; 19 Pharmacy Unit, Mater Domini Hospital, 88100 Catanzaro, Italy; adele.defrancesco@materdominiaou.it; 20 Thoracic Oncology Unit, IRCCS Istituto Romagnolo per lo Studio dei Tumori (IRST) “Dino Amadori”, 47014 Meldola, Italy; lucio.crino@irst.emr.it (L.C.); angelo.delmonte@irst.emr.it (A.D.)

**Keywords:** non-small cell lung cancer, immunotherapy, retrospective study, real-world data

## Abstract

**Simple Summary:**

Pembrolizumab monotherapy remains the first-line standard of care for patients with metastatic NSCLC and a PD-L1 tumor proportion score ≥ 50%. However, although other real-world studies have been published, long-term follow-up data on progression-free survival, overall survival, treatment response, and safety in a large cohort of patients are still lacking. We evaluated these outcomes in a cohort of 880 patients treated with first-line pembrolizumab monotherapy in 16 Italian centers. We also analyzed the prognostic impact of variables such as age, sex, histology, PD-L1 expression, ECOG, habitual smoking, and the presence of brain metastases. Median PFS and OS were 8.6 and 25.5 months, respectively, consistent with the results of *Keynote-024*. According to univariate analysis, it was determined that PD-L1 expression, ECOG, and habitual smoking had an impact on PFS, while age, sex, ECOG, histology, and habitual smoking had an impact on OS. However, results from univariate analysis should be considered with caution.

**Abstract:**

Results from the phase III *Keynote-024* clinical trial established pembrolizumab monotherapy as the first-line standard of care for patients with metastatic NSCLC who have PD-L1 expression ≥ 50%, *EGFR*, and *ALK* wild-type tumors. However, given the differences between patients treated in routine clinical practice and those treated in a clinical trial, real-world data are needed to confirm the treatment benefit in standard practice. Given the lack of data on large cohorts of patients with long follow-ups, we designed an observational retrospective study of patients with metastatic NSCLC who were treated with pembrolizumab, starting from its reimbursement eligibility until December 2020. The primary endpoints were PFS and OS, determined using the Kaplan–Meier method. Response and safety were also evaluated. We followed 880 patients (median follow-up: 35.1 months) until February 2022. Median PFS and OS were 8.6 months (95% CI: 7.6–10.0) and 25.5 months (95% CI: 21.8–31.6), respectively. We also found that ECOG PS, PD-L1 expression, and habitual smoking were prognostic factors for PFS, while age, sex, ECOG PS, habitual smoking and histology had an impact on OS. Multivariable analysis confirms the prognostic role of PD-L1 for PFS and of ECOG for both PFS and OS. 39.9% of patients reported an adverse event, but only 6.3% of patients discontinued therapy due to toxicity. Our results suggest a long-term benefit of pembrolizumab in the first-line setting, as well as a safety profile consistent with the results of *Keynote-024*. Many collected variables appear to influence clinical outcome, but results from these exploratory unadjusted analyses should be interpreted with caution.

## 1. Introduction

In 2023, about 44,000 new cases of lung cancer are expected in Italy: it is the second most common neoplasm in men (15%) and the third most common in women (6%). In 2022, about 35,700 deaths from lung cancer were recorded in Italy: 23,600 in men and 12,100 in women. Lung cancer is the first cause of cancer death in men and the second in women. The 5-year survival rate of lung cancer patients in Italy is 16% for men and 23% for women, which is negatively affected by the high proportion of patients diagnosed with advanced-stage disease [1].

Nevertheless, there have been significant advancements in therapeutic options for patients with metastatic non-small cell lung cancer (NSCLC) in recent years, which provides hope for improving survival rates. Developments in molecular testing and the availability of new therapies targeting specific molecular alterations have significantly impacted patient management. Clinicians are now able to select therapies that are most likely to improve individual clinical outcomes, taking into account patients’ characteristics and tumor histology. 

According to several studies, it has been found that a significant percentage of NSCLC patients, ranging from 28–44%, possess biomarkers that can be targeted by first-line (1L) or subsequent therapies approved for metastatic NSCLC [2,3,4,5,6]. Currently, targetable genetic alterations for NSCLC include epidermal growth factor receptor (*EGFR*), anaplastic lymphoma kinase (*ALK*), ROS proto-oncogene 1 (*ROS-1*), Kirsten rat sarcoma virus (*KRAS*), B-Raf proto-*oncogene* (*BRAF*), MET proto-oncogene (*MET*), human epidermal growth factor receptor (HER2), neurotrophic tyrosine receptor kinase (*NTRK*), and the rearranged during transfection (*RET*) gene [7]. In addition to molecularly targeted therapies, immunotherapies have also improved treatment for non-small cell lung cancer (NSCLC), providing survival benefits for patients with metastatic NSCLC regardless of their PD-L1 expression level [8].

For patients with metastatic NSCLC without driver mutation of *EGFR*, *ALK*, and *ROS1*, drugs that act on the PD-1/PDL-1 axis are predominantly used in the first-line setting with or without platinum chemotherapy. This happened since pembrolizumab monotherapy proved its superiority in terms of progression-free survival (PFS) (10.3 mo. vs. 6.0 mo., HR 0.50; 95% CI, 0.37 to 0.68; *p* < 0.001) and overall survival (OS) (30.0 mo. vs. 14.2 mo.; HR, 0.63; 95% CI, 0.47 to 0.86; *p*-value = 0.002) against platinum-based chemotherapy in *Keynote-042* and become available and reimbursed by the national health system (NHS) in June 2017 for patients with NSCLC with a tumor proportional score (TPS) of PDL-1 ≥ 50% [9,10]. Following the results of *Keynote*-*189* and *Keynote*-*407*, pembrolizumab has become available, in combination with chemotherapy selected based on tumor histology, for non-squamous and squamous cancers since December 2019 and December 2020, respectively [11,12]. However, the Italian Regulatory Agency (AIFA) limited reimbursement for chemo-immunotherapy to patients with PD-L1 expression < 50%, even though its superiority over chemotherapy alone was demonstrated regardless of PD-L1 expression. Considering this limitation, single-agent immunotherapy is still the best available option for previously untreated patients with metastatic NSCLC with a tumor proportional score (TPS) of PDL-1 ≥ 50% and without *EGFR*, *ALK*, and *ROS1* mutation [13]. 

The survival benefit shown by pembrolizumab is also confirmed by long-term data, which are not yet available for the other two alternatives, Atezolizumab and Cemiplimab, which have been reimbursed by the NHS for the same therapeutic indication since June and August 2022, respectively. Nevertheless, the role of real-world observational studies is very important to confirm that the benefits seen in clinical trials are also seen in the population treated in everyday clinical practice.

Velcheti et al. recently published the 3-year follow-up real-world results of their observational study in US oncology practice. The study has confirmed the results of the Keynote-024 clinical trials in terms of real-world time on treatment (rwToT), which is a surrogate indicator associated with survival in NSCLC studies [14].

However, given the differences in the population that could be treated in Italy for regulatory reasons, the availability of different therapeutic options for subsequent lines that could change patient survival, and the importance of outcome indicators such as progression and death of patients treated in real-life, we designed a multicenter observational study to evaluate the long-term effectiveness and safety outcome of pembrolizumab monotherapy (the *PEMBROREAL* study).

This work aims to show the 3-year effectiveness and safety results from a cohort of patients treated in sixteen institutions in Italy following the standard clinical practice. 

## 2. Materials and Methods

### 2.1. Study Design

PEMBROREAL is a retrospective, multicenter study conducted in Italy. The study involved a cohort of patients who received at least one dose of pembrolizumab monotherapy in the first-line treatment of metastatic NSCLC without EGFR and ALK driver mutation. The medical records of adult patients with NSCLC who were consecutively treated at one of the sixteen study centers were reviewed by study investigators from each participating center, according to the eligibility criteria established by AIFA. For each enrolled patient, chart extractions were performed on two predetermined dates: one in January 2021 and the other in February 2022. The first data extraction aimed to guarantee that participating centers met high standards in collecting the data. In contrast to the eligibility criteria for *Keynote-024*, it was decided that patients with NSCLC who have not received prior treatment for metastatic disease will also have access to pembrolizumab monotherapy if they have ECOG PS 2, active central nervous system (CNS) metastases, and a life expectancy of less than three months. To be considered for inclusion, patients who began treatment with pembrolizumab between June 2017 and December 2020 were required to have provided informed consent for their medical records to be accessed. Patients who could not be reached despite all reasonable efforts or who had passed away were included, in accordance with national regulations. Patients who received pembrolizumab through an interventional clinical trial, compassionate-use program, or off-label were not eligible for inclusion. The study protocol (protocol approval ID: 6346/2020 I.5/187) was approved by the Ethics Committees of all participating institutions. The study was conducted in accordance with the principles of the 1964 Declaration of Helsinki and its subsequent amendments.

### 2.2. Assessment

The study aimed to evaluate the effectiveness, safety, and activity of pembrolizumab monotherapy in previously untreated metastatic NSCLC patients, based on the reimbursement criteria set by AIFA. The aim was to assess the consistency of the results obtained in Keynote-042 in real-world clinical practice. The study evaluated two main outcomes: real-world PFS and OS. PFS was calculated starting from the date of first administration of pembrolizumab (index date) until the date of progression as determined by the treating physician, death (if no progression), or the end of follow-up, whichever occurred first. OS was calculated from the index date until death or the end of follow-up, whichever occurred first. Additionally, we assessed the overall response rate (ORR), calculated as the proportion of patients who achieved a complete or partial response out of the total number of patients. Similarly, the disease control rate (DCR) was determined by the proportion of patients who achieved a complete or partial response or disease stability out of the total number of patients. Duration of response (DoR) is measured from the date of the first documented response until disease progression or death (if no progression). During the follow-up period, patients who did not exhibit any signs of progression were censored. The identification and management of adverse events (AEs) were carried out by the treating physician. The grade of AE was determined by the reporter using the Common Terminology Criteria for Adverse Events, version 4. Due to the real-world nature of *PEMBROREAL*, physicians may determine progression and response to therapy through assessments or by following the Response Evaluation Criteria in Solid Tumours (RECIST) version 1.1, depending on local practice.

In addition, key secondary endpoints include rwPFS and OS for subgroups of interest, as well as demographic, patient, and disease characteristics.

### 2.3. Statistical Analyses

No formal sample size calculation was performed. Categorical variables were presented as absolute and relative frequency, whereas for continuous variables, data were presented as median with minimum and maximum values. Patients lost to follow-up (i.e., alive at last visit or contact before database cut-off) were censored from rwPFS and rwOS data (i.e., alive at last visit or contact before database cut-off). Median and landmark rates were calculated using the Kaplan–Meier method, and the corresponding 95% confidence interval was calculated using Greenwood’s method. The potential prognostic role of the investigator-identified factors was assessed using the log-rank test. A multivariable Cox regression model was used for the assessment of independent prognostic factors, and the hazard ratios (HR) were calculated. Proportional hazard assumptions were tested using Schoenfeld residuals. In case of violation, a time-dependent effect was also calculated and added to the main effect.

Analyses were based on the total population involved in the study. A complete case analysis was conducted without any imputation, due to the retrospective and pragmatic nature of the study, as well as the unlikelihood of finding a reliable pattern derived from other variables in a multiple imputation context; subgroup analyses (i.e., stratified by disease, therapy, or other demographic or prognostic variables) were performed as indicated by the investigators at the participating centers. A *p*-value < 0.05 was considered statistically significant. Statistical analyses were performed using STATA/MP 15.0 for Windows (StataCorpLP, College Station, TX, USA).

## 3. Results

### 3.1. Patients and Treatment Characteristics

Between 25 June 2017 and 31 December 2020, a total of 880 eligible patients received their first pembrolizumab dose and were included in the analysis. The median follow-up duration in the full analysis set was 35.1 months (ranging from 0.1 to 56.0). Only 39 patients (4.4%) were lost to follow-up, and none of the enrolled patients were excluded from the final analysis. The median age of patients in the full analysis set was 69.9 years (ranging from 37.9 to 90.2) at their first administration of pembrolizumab; 229 (26.0%) were aged above 75 years. The majority of patients (70.2%) were male and had a PS of 0 or 1 (91.9%) at their first administration of pembrolizumab. The most common histological type was lung adenocarcinoma, with 673 (76.5%) patients enrolled, while 158 (18.0%) patients had squamous cell carcinoma. In addition to the two most prevalent histological types, our study included a small number of patients with NSCLC not otherwise specified (NOS), adenosquamous carcinoma, and large-cell lung cancer. For one patient, the tumor histology was unknown.

The TPSs of PD-L1 were immunohistochemically evaluated by a validated 22C3 IHC laboratory test for all patients. All patients enrolled had PD-L1% expression ≥ 50%. For most of them (527 of the 880 patients (59.9%)), the exact expression of PD-L1 was available in the clinical documentation. Of these 527 patients, 120 (22.8%) had high expression of PD-1 (≥90%). A total of 134 patients were diagnosed with brain metastasis (15.7%). For 29 patients included in the study, the presence or absence of brain metastasis was not documented. Out of the 880 patients included in the study, 542 (61.6%) had known smoker status, with the majority being either current (n = 186; 34.3%) or former smokers (n = 251; 46.3%). *EGFR* and *ALK* mutations were tested for 788 and 790 of 880 included patients, respectively, using next-generation sequencing (NGS) for the first mentioned mutation and immunohistochemical validated Ventana *ALK* (D5F3) and rabbit monoclonal antibody for the second one. The result was negative for 100.0% of the tested patients. Of note, all patients with unknown *EFGR* and *ALK* status, 92 and 90, respectively, had a squamous NSCLC (Table 1). Giving the rarity of these two mutations and the low efficacy of target treatments in this histological subtype cell tumor, searching for these driver-gene alterations is not mandatory considering current guidelines and the eligibility criteria for NHS reimbursement. 

The study did not collect data on other driver mutations, such as KRAS, BRAF, RET, and ROS1, which may have affected OS and PFS. It is worth noting that participating centers evaluated these mutations at their discretion.

Additionally, the median total treatment time, including dose interruptions, was 189 days. Notably, 34.7% of patients received pembrolizumab for more than 12 months. Patients received a median of 9.0 infusions of pembrolizumab (range: 1–75). It is worth noting that 8.1% of patients had a temporary treatment interruption of more than three weeks for any reason.

### 3.2. Reasons for Discontinuing Pembrolizumab

The most common reasons for discontinuing pembrolizumab treatment were disease progression (53.9%) and death (13.6%). A small percentage of patients (6.3%) stopped treatment due to unacceptable toxicity. At the time of data cut-off, 16.0% of patients were still receiving pembrolizumab treatment (refer to Supplementary Table S1).

### 3.3. Analysis of Real-World PFS

**Figure 1:**
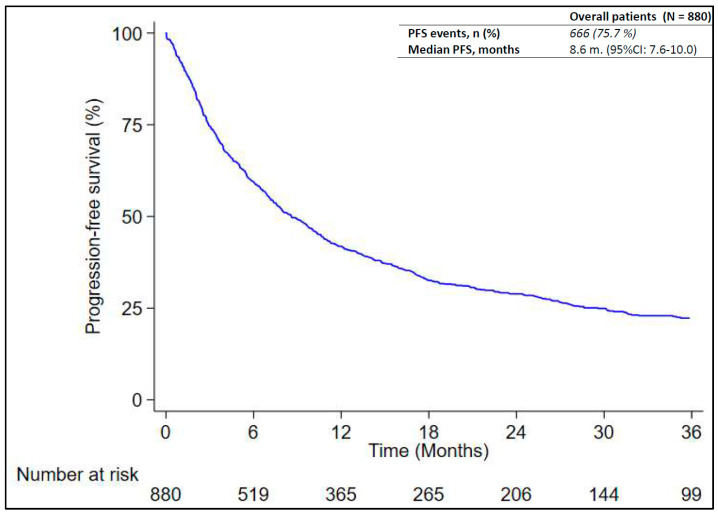
Progression-free survival of patients, shown as Kaplan–Meier distribution.

At data cut-off, 666 out of 880 patients (75.7%) experienced disease progression or died with no documentation of disease progression. Progression was determined per RECIST or clinical assessment. Radiological assessment was performed in local clinical practice, usually every three months, but this was not always possible for logistical or clinical reasons. The median RwPFS was 8.6 months (95% CI: 7.6–10.0) as reported in Figure 1. According to Table 2, patients with PD-L1 ≥ 90% had a significantly longer RwPFS (median: 13.9 months; 95% CI: 8.9–19.0) compared to those with PD-L1 between 50% and 69% (median: 6.9 months; 95% CI: 5.4–8.9) and patients with PD-L1 level between 70% and 89% (median: 7.0 months; 95% CI: 5.6–10.0; *p*-value = 0.032) (Figure S1A in Supplementary Materials). The study found that patients with an ECOG performance status of 0 had a median survival time of 11.1 months (95% CI: 9.8–14.8), which was longer than those with a performance status of 1 and 2, who had median survival times of 7.8 months (95% CI: 6.4–9.5) and 2.8 months (95% CI: 1.7–5.1), respectively (*p* = 0.013) (refer to Figure S1B in Supplementary Materials). Another significant difference in rwPFS was registered between patients who were smokers and former smokers (median: 10.8 months; 95% CI: 8.9–13.1) and patients who never smoked (median: 5.9 months; 95% CI: 3.9–8.6; *p*-value = 0.007) (Figure S1C Supplementary Materials).

Meanwhile, RwPFS was not different among patients aged less than 75 (median: 8.5 months; 95% CI: 7.1–10.1) versus those aged greater or equal to 75 years (median 9.1 months; 95% CI: 7.4–10.8; *p*-value = 0.640), nor was RwPFS different among patients with a squamous (median: 8.4 months; 95% CI: 6.2–12.1) and non-squamous (median: 8.6 months; 95% CI: 7.5–10.4; *p*-value: 0.092) cancer type. Patients’ sex also appears to be unrelated to RwPFS, although it was slightly longer in female patients (median: 9.2; 95% CI: 7.2–12.1) than in male patients (median: 8.3 months; CI 95%: 7.2–10.0; *p*-value 0.108). Central nervous system involvement did not have a significant effect on rwPFS, but it was shorter in patients with brain metastases (median: 6.3; 95% CI: 4.2–8.9) than in those without brain metastases (median: 9.3 months; CI 95%: 7.7–10.4; *p* = 0.053).

The multivariable analysis in Supplementary Table S4 confirms that differences in rwPFS between subgroups stratified by PD-L1 level were mainly driven by differences between patients with PD-L1 ≥ 90%. These patients had a lower risk of progression compared to patients with PD-L1 levels greater than 50% and less than or equal to 69% (HR. 0.62; 95% CI: 0.46–0.83; *p*-value = 0.001) than the difference between the patients in this latter group and patients with a PD-L1 level greater than 70% and less than or equal to 89% (HR. 0.82; 95% 0.64–1.05; *p*-value = 0.125). Patients with PS ECOG 1 and with PS ECOG 2 had a higher risk of progression compared to patients with PS ECOG 0, with an HR equal to 1.51 (95% CI:1.20–1.89; *p*-value < 0.001) and 2.36 (95% CI: 1.50–3.69; *p*-value < 0.001), respectively. The difference between smoker or former smoker and current smoker in rwPFS was not confirmed by the multivariable model (HR 0.81; 95% CI: 0.61–1.07; *p* = 0.144). 

### 3.4. Analysis of Real-World OS

**Figure 2:**
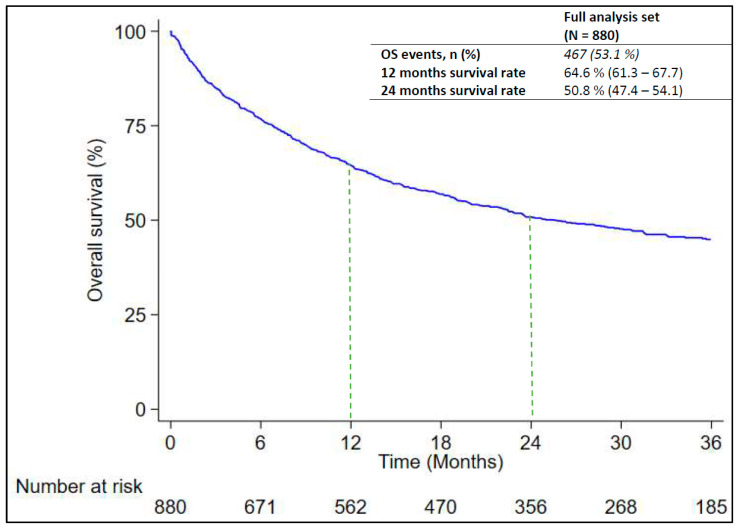
Overall survival of patients, shown as Kaplan–Meier distribution. Dashed lines represent 12- and 24-month landmark analyses.

At the time of the data cut-off, 467 out of 880 patients (53.1%) died. The median RwOS was 25.5 months (95% CI: 21.8–31.6) as reported in Figure 2. The RwOS was analyzed in subgroups of interest to evaluate any possible associations with prognostic factors. As found in Table 3, RwOS was significantly longer among female patients (median: 39.1 months; 95% CI: 25.5–NE) versus male patients (median: 22.3 months; 95% CI: 17.8–28.9; *p*-value = 0.014) and in patients with non-squamous NSCLC (median: 18.5 months; 95% CI: 15.5–22.8) than in patients with squamous NSCLC (median: 29.9 months; 95% CI: 23.5–39.1; *p*-value = 0.010). However, the effect of the latter variables seems to be time-dependent and resulted only when considering the survival rate after 24 months of observation with non-squamous NSCLC versus squamous NSCLC (53.2%; CI: 49.4–56.8 and 40.4%; CI: 32.5–48.2, respectively) as compared to the difference in survival rate after 12 months of observation (64.7%; CI: 61.1–68.1 and 64.6%; CI: 56.6–61.5, respectively). Moreover, a significant difference in RwOS was found among patients ≥ 75 years (median: 15.4 months; 95% CI: 12.4–22.5) and patients < 75 years (median: 30.7 months; 95% CI: 23.6–48.7; *p*-value = 0.002) and among patients who were current or former smokers (median: 22.4 months; 95% CI: 18.7–28.9) and those that never smoked (median: N.R.; *p*-value = 0.044). Finally, survival also appears to be influenced by ECOG PS, considering the difference found among patients with ECOG PS 0 (median: 38.5 months; 95% CI: 29.5–N.E.), ECOG PS 1 (median: 18.6 months; 95% CI: 14.1–18.6), and ECOG PS 2 (median: 5.9 months; 95% CI: 2.8–15.5; *p* < 0.001) (Figure S2A–E in Supplementary Materials).

PD-L1 expression and the presence of brain metastases appear to be unrelated with RwOS, considering that no differences were found between patients with (median: 23.5 months; 95% CI: 13.5–29.9) and without brain metastases (median: 25.5 months; 95% CI: 20.0–35.7; *p*-value = 0.291) and among patients with PD-L1 greater than or equal to 90% (median: 22.5 months; 95% CI: 14.1–40.4) versus patients with PD-L1 between 50% and 69% (median: 15.5 months; 95% CI: 10.7–19.8) and patients with a PD-L1 level between 70% and 89% (median: 15.3 months; 95% CI: 11.6–21.3, *p*-value = 0.117).

The multivariate analysis is shown in Supplementary Table S5: among all the prognostic factors that affected RwOS, the only variable that confirmed this effect was ECOG PS, and the effect was time-dependent.

### 3.5. Analysis of Treatment Response

In the study, it was found that out of 880 patients, 611 (69.4%) reported a response to treatment. Among these patients, 179 (20.3%) had a partial (17.6%) or complete response rate (2.7%) as the best response to treatment (refer to Supplementary Table S2). At the first evaluation, 25.9% of patients had experienced disease progression. The disease control rate (DCR) for the included patients was 79.2%. The median duration of response (DoR) for patients with a documented complete or partial response was 27.1 months (95% CI: 22.0–33.8). 

### 3.6. Safety

Out of 880 eligible patients, 351 patients (39.9%) reported at least one AE. AEs reported for at least 15% of the eligible patients included the following: gastrointestinal disorders (23.8%); general disorders and administration site conditions (23.4%); skin and subcutaneous tissue disorders (22.4%); respiratory, thoracic, and mediastinal disorders (15.9%). 

A total of 67 patients reported grade 3–4 AEs, of which 14 were gastrointestinal disease, 13 were skin and subcutaneous tissue disorders, 13 were general disorders and administration site conditions, and 13 were related to alteration of diagnostic exams (Table 4). 

It is worth noting that most of the reported adverse events did not require any management measures such as drug administration suspension, reduction, definitive interruption, hospitalization, or specific pharmacologic treatment. Among the patients who experienced an adverse event (AE) that required specific measures, it was observed that 20.1% (177 cases) were managed with pharmacologic treatment, while 8.1% (71 cases) required temporary treatment interruption. It was found that only 5.3% (47 patients) definitively discontinued treatment due to toxicity (see Supplementary Table S3).

## 4. Discussion

In this large multicenter, real-life observational study, we considered 880 patients with metastatic NSCLC and a PD-L1 expression ≥ 50%, *EGFR*, and *ALK* wild-type tumors who were treated with first-line pembrolizumab monotherapy. With a median follow-up time of 35.1 months, we reported a median PFS of 8.6 months and a median OS of 25.5 months. These results are consistent with findings from *Keynote-024*, in which PFS was 7.7 months (CI 95%: 6.1–10.2) and OS was 26.3 months (CI 95%: 18.3–40.4). However, we observed that patients were somewhat older in our cohort of patients than in the experimental arm of *Keynote-024* (median age 69.9 vs. 65.5) and included proportionately more women (40.3% vs. 29.8%).

In addition, we included 8.1% of patients with ECOG PS 2 with less than three months of life expectancy and with active brain metastases who were treated in routine clinical practice but would be excluded from enrollment in a clinical trial. Moreover, one of the limitations of *Keynote-024* was that only a few patients who never smoked were enrolled (3.2%), whereas in the real-world setting, this rate was higher among patients whose smoking habits were known (19.4%). 

To the best of our knowledge, many other real-world studies that have been published had shorter follow-ups, focused on surrogate endpoints, or used results that referred to few patients. We have compiled a table of all previously published real-world studies of the same treatment for similar populations. (Table 5) [14,15,16,17,18,19,20,21,22,23,24,25,26,27,28,29,30]. Compared with the results of other previously published real-world studies in the same setting, OS in the PEMBROREAL study was longer, based on a longer follow-up, and derived from the observation of more patients than those in the majority of other reported studies. PFS was similar to that previously observed in the reported studies, except for that of Jiménez Galán [16], in which it should be noted that almost one third of the patients enrolled had an ECOG PS ≥ 2. A possible explanation of the longer OS in our recent study could be the availability of newer second-line therapies, especially for patients with mutations of other driver genes (e.g., *KRAS*, *BRAF*, *RET*, *MET*), that could be responsible for a longer survival for these patients. On the other hand, the consistency of the PFS data could be explained by the fact that the availability of this outcome takes less time if compared to OS, and therefore, data from other studies are sufficiently robust, despite the limited follow-up of some of them.

Univariate Cox regression confirmed that PD-L1 expression, ECOG PS, and smoker status significantly influence PFS. Other variables studied, such as age ≥ 75 years at diagnosis, sex, histological type, and presence of brain metastases, were not statistically significant in determining PFS. However, a slightly favorable trend for patients without brain metastases was observed for this latter variable. 

According to previously published papers [15,16,31,32], clinical outcomes are significantly improved in NSCLCs with a PD-L1 expression of ≥90% and ECOG PS < 2. The prognostic role of ECOG PS was explored in many other studies, and despite the limitations due to the subjective nature of this parameter—considering that two physicians may classify the same patient with a different performance status—it is an important variable influencing patient outcome. In addition, we know from the work of Facchinetti et al. that the worst outcomes were found for patients for whom a high ECOG PS value was determined by disease burden rather than for patients for whom it was determined by comorbidities [33]. However, it was not possible to differentiate between these two types of patients in our study. 

In NSCLC patients receiving immunotherapy, smoking status has previously been shown to be associated with clinical outcomes [29]. A recent study of patients with NSCLC treated with different drugs active on the PD-1/PD-L1 axis across multiple lines showed a non-statistically significant trend towards an improved PFS in heavy and light smokers compared to those who never smoked [34]. The authors found that patients who smoked heavily had a higher tumor mutational burden (TMB) in their tumors, which makes cancer cells easier for the immune system to recognize; this was an explanation for the difference in the clinical outcome. Notably, the difference between patients who never smoked and former or current smokers was not confirmed by the multivariable model, so this finding on the prognostic role of smoking habit should be considered with caution.

Interesting results were obtained from the univariate Cox regression analysis of the variables that influence OS. Not surprisingly, a significantly better OS was found for patients with age at diagnosis < 75 years and for patients with lower value of ECOG PS. We considered more interesting the role of sex in determining OS, since, in our results, female patients had a better prognosis than male patients. Another interesting variable of prognostic significance was tumor histology, since patients with a non-squamous tumor type had a better outcome than patients with a squamous tumor type. Finally, the prognostic value determined for smoker status was inverted if we considered OS compared to PFS. If the outcome for PFS was better for patients who were former or current smokers, the outcome for OS was better for patients who had never smoked. It is important to note that these findings are exploratory and unadjusted; therefore, they should be interpreted with caution.

Research has consistently shown that sex is a prognostic factor in lung cancer, even before the availability of immune checkpoint inhibitors. A recent study conducted on a large Australian cohort confirmed that women have a significantly longer survival rate after a lung cancer diagnosis compared to men [35]. The study also found that men were typically older at diagnosis, less likely to be non-smokers, and had more comorbidities. Some authors have suggested that lung cancer in women may have a different natural history, which could be related to immunological differences as well as patient factors [36,37].

Regarding results about the influence of cancer histology on OS, these were consistent with findings from the real-world study conducted by Tambo et al. [26]. In this study, patients with squamous NSCLC had a greater number of metastatic sites compared with patients with adenocarcinoma, and these findings were correlated with a longer OS. Unfortunately, there are no published data relative to the effect of histologic type on the outcome of patients treated with immunotherapy; however, squamous cell carcinoma is a well-known poor prognostic factor.

Considering the opposite effect of smoker status on OS, these results are consistent with findings of the meta-analyses conducted by Popat et al. [38] and could be explained by the fact that, according to the previously published report, patients who were never smokers were more likely to be women and be diagnosed with non-squamous tumor histology [39].

Treatment response seems to be inconsistent across different studies. In our cohort of patients, ORR results were lower if compared with results from *Keynote-024* (ORR: 44.8% vs. 20.3%). However, these differences may be explained by differences in response assessment between an experimental clinical trial, where the response is rigorously assessed and centrally reviewed, and retrospective observational studies, where reports of tumor response are individually reported by a single clinician and collected by chart extraction, with concerns about missing reports.

Safety assessment revealed that pembrolizumab monotherapy was generally well tolerated. Comparing the results of our observational study with those of patients treated in the experimental arm of *Keynote-024*, the rate of patients with at least one adverse event was halved in patients treated in current clinical practice (39.9 vs. 73.4). Grade 3 and 4 AE were uncommon in current clinical practice, occurring only in 67 out of 880 (7.6%) patients. Interestingly, in the majority of cases, toxicity management do not require specific intervention, and situations that do require specific management can be easily managed with pharmacological therapy or temporary withholding of pembrolizumab.

Most concerns regarding safety evaluation in our study are related to the phenomenon of under-reporting of toxicity by treating physicians, especially in the case of low-grade AEs or for those considered as not clinically relevant.

Strengths of this study include the large multicenter cohort with the longest follow-up ever published. These considerations provide consistency to our outcome results compared with previous publications with a limited follow-up or with few patients.

However, it is important to note that retrospective data evaluation has limitations such as the potential for missing or inaccurately recorded data. For example, smoking status was unknown in more than 23% of patients, and for 40% of patients, we only know that they had a TPS of PD-L1 ≥ 50%, but we did not know the exact value of their score. However, we have no reason to think that missing data for some of the variables considered for univariate or multivariate analysis could be unbalanced between the subgroups.

Another limit is related to outcome assessments. Patients included in our study were treated within the valid standard of care outside a clinical trial with varying imaging intervals, thereby potentially biasing PFS. Additionally, progression can be determined based on radiological or clinical evidence. It may be worthwhile to conduct further analysis to explore the potential impact of this limitation on PFS.

Finally, considering that 13 of the 16 institutions selected to participate in our study had an internationally recognized role in cancer research and treatment, this could be considered a selection bias in the choice of patients, as these patients are more likely to receive better care than patients treated at less prestigious hospital sites, resulting in better outcomes.

Despite these limitations, our results are consistent with those of other retrospective studies and with the results of *Keynote-024*, and they help to identify patients who may best benefit from treatment with pembrolizumab monotherapy.

## 5. Conclusions

In conclusion, the results of our retrospective observational study of pembrolizumab monotherapy in first-line metastatic NSCLC with PD-L1 ≥ 50% are consistent with previously reported real-world studies and the results of *Keynote-024.*

This is true even though the distribution of patient characteristics in routine clinical practice is slightly different from that in the clinical trial and even though patients in clinical trials are selected by strict selection criteria, including some related to performance status, presence of active brain metastases, and life expectancy.

In addition, we identified several prognostic factors that influence patient outcomes. This is of interest for patients, who should be informed about the benefit of a particular therapy for patients with their characteristics; for physicians, who can identify which patients will benefit most from a particular treatment and which will not; and to health care administrators, who want to understand whether a new technology is cost-effective or not.

## Figures and Tables

**Table 1:** Patients’ demographic and disease characteristics.

Characteristics	N° of Patients n = 880
** Age (continuous), median (minimum-maximum) **	69.9 (37.9–90.2)
** Age (categorical), n (%) **	
<75	651 (74.0)
≥75	229 (26.0)
**Sex, n (%)**	
Male	618 (70.2)
Female	262 (29.8)
** Histology, n (%) **	
Adenocarcinoma	673 (76.5)
NOS carcinoma	37 (4.2)
Large-cell lung Cancer	4 (0.5)
Adenosquamous	7 (0.8)
Squamous	158 (18.0)
Unknown—N/D	1
**EGFR status known, n (%)**	
Yes	788 (89.5)
No	92 (10.5)
**EGFR mutation, n (%)**	
Negative	788 (100.0)
**ALK status known, n (%)**	
Yes	790 (89.8)
No	90 (10.2)
**ALK status known, n (%)**	
Negative	790 (100.0)
** PDL1, n (%) **	
50–59%	140 (26.5)
60–69%	72 (13.7)
70–79%	104 (19.8)
80–89%	91 (17.3)
90–99%	104 (19.7)
100%	16 (3.0)
Unknown	353
** PS ECOG, n (%) **	
0	366 (42.0)
1	435 (49.9)
2	71 (8.1)
Unknown	8
** Smoking history, n (%) **	
Ex smoker	251 (46.3)
Smoker	186 (34.3)
Not smoker	105 (19.4)
Unknown	338
** Presence of brain metastases, n (%) **	
Yes	134 (15.7)
No	717 (84.3)
Unknown	29

Note: Percentages reported in the table were calculated using the number of patients with available data (for each variable).

**Table 2:** Univariable analysis of real-world progression-free survival.

Variable	Number of Patients	Number of Events	Median Rw-PFS (95% CI)	*p*-Value (Log-Rank Test)
All cases	880	666	8.6 (7.6–10.0)	-
**Age**				
<75 yrs	651	488	8.5 (7.1–10.1)	0.640
≥75 yrs	229	178	9.1 (7.4–10.8)	
**Sex**				
Male	618	480	8.3 (7.2–10.0)	0.108
Female	262	186	9.2 (7.2–12.1)	
**Histologic types**				
Squamo	158	133	8.4 (6.2–12.1)	0.092
No squamo	721	532	8.6 (7.5–10.1)	
**PDL1**				
50–69%	212	176	6.9 (5.4–8.9)	0.032
70–89%	195	155	7.0 (5.6–10.0)	
≥90%	120	79	13.9 (8.9–19.0)	
**PS ECOG**				
0	366	256	11.1 (9.8–14.8)	<0.001
1	435	339	7.8 (6.4–9.5)	
2	71	64	2.8 (1.7–5.1)	
**Smoking history**				
Never smoker	105	86	5.9 (3.9–8.6)	0.007
Smoker or former smoker	437	317	10.8 (8.9–13.1)	
**Presence of brain metastases**				
Yes	134	108	6.3 (4.2–8.9)	0.053
No	717	538	9.3 (7.7–10.4)	

**Table 3:** Univariable analysis of real-world overall survival.

Variable	Number of Patients	Number of Events	Median Rw-OS (95% CI)	12-Months Rw-OS (95% CI)	24-Months Rw-OS (95% CI)	*p*-Value Log-Rank Test
All cases	880	467	25.5 (21.8–31.6)	64.6 (61.3–67.7)	50.8 (47.4–54.1)	-
**Age**						
<75 yrs	651	326	30.7 (23.6–48.7)	66.8 (63.0–70.3)	53.7 (49.7–57.6)	0.002
≥75 yrs	229	141	15.4 (12.4–22.5)	58.3 (51.6–64.4)	42.8 (36.2–49.3)	
**Sex**						
Male	618	347	22.3 (17.8–28.9)	62.9 (59.0–66.6)	48.1 (44.1–52.1)	0.014
Female	262	120	39.1 (25.5-NE)	68.5 (62.5–73.8)	57.2 (50.8–63.1)	
**Histologic types**						
Squamo	158	101	18.5 (15.5–22.8)	64.6 (56.6–71.5)	40.4 (32.5–48.2)	0.010
No squamo	721	365	29.9 (23.5–39.1)	64.7 (61.1–68.1)	53.2 (49.4–56.8)	
**PDL1**						
<50–69%	212	138	15.5 (10.7–19.8)	54.7 (47.8–61.1)	39.3 (32.6–45.9)	0.117
70–89%	195	131	15.3 (11.6–21.3)	56.9 (49.7–63.5)	38.4 (31.4–45.4)	
≥90%	120	66	22.5 (14.1–40.4)	66.5 (57.2–74.2)	49.4 (39.9–58.2)	
**PS ECOG**						
0	366	171	38.5 (29.5-NE)	73.9 (69.1–78.1)	59.1 (53.7–64.0)	<0.001
1	435	241	18.6 (14.1–28.6)	60.4 (55.6–64.8)	47.2 (42.4–51.9)	
2	71	51	5.9 (2.8–15.5)	42.3 (30.7–53.4)	29.3 (18.8–40.5)	
**Smoking history**						
Never smoker	105	48	NR	69.5 (59.8–77.4)	58.6 (48.5–67.4)	0.044
Smoker or former smoker	437	246	22.4 (18.7–28.9)	64.6 (59.9–68.9)	48.1 (43.2–52.9)	
**Presence of brain metastases**						
Yes	134	78	23.5 (13.5–29.9)	60.5 (51.6–68.2)	48.8 (39.8–57.1)	0.291
No	717	377	25.5 (20.0–35.7)	65.2 (61.6–68.6)	50.9 (47.2–54.6)	

NR = not reached; NE = not estimable from statistical software.

**Table 4:** Adverse reactions reported.

Toxicities	N° Patients n = 880 (%)	
At Least One Adverse Reaction	351 (39.9%)	
	Any Grade	Grade 3–4
Infections and infestations	22 (2.5)	1 (0.1)
Blood and lymphatic system disorders	74 (8.4)	6 (0.7)
Immune system disorders	5 (0.6)	0 (0.0)
Endocrine disorders	60 (6.8)	0 (0.0)
Metabolism and nutrition disorders	92 (10.5)	2 (0.2)
Psychiatric disorders	13 (1.5)	0 (0.0)
Nervous system disorders	48 (5.5)	0 (0.0)
Eye disorders	13 (1.5)	0 (0.0)
Cardiac disorders	23 (2.6)	1 (0.1)
Vascular disorders	12 (1.0)	0 (0.0)
Respiratory, thoracic, and mediastinal disorders	140 (15.9)	1 (0.1)
Gastrointestinal disorders	209 (23.8)	14 (1.6)
Hepatobiliary disorders	14 (1.6)	0 (0.0)
Skin and subcutaneous tissue disorders	197 (22.4)	13 (1.5)
Musculoskeletal and connective tissue disorders	90 (10.2)	2 (0.2)
Renal and urinary disorders	15 (1.7)	1 (0.1)
General disorders and administration site conditions	203 (36.6)	13 (1.5)
Diagnostic exams	89 (10.1)	13 (1.5)

**Table 5:** Real-world studies previously published for patients treated for metastatic NSCLC PD-L1 ≥ 50% ALK and EGFR wild-type tumors with pembrolizumab monotherapy.

Paper, Publication Year	Patients Number	Nations Involved	Median Follow Up	Median Overall Survival	Median Progression Free Survival	Others Surrogate Endpoints Evaluated	Note
[14] Velcheti V; February 2022	1044	USA, multicentric	34 months	/	/	rwTOT = 7.4 months (95%CI: 6.3–8.1)	
[15] Bérard G; March 2023	279	Canada (Quebec), multicentric	7.53 months	17.3 months (95% CI: 12.9–NR)	9.4 months (95% CI: 6.6–11.2)	/	2 patients with PD-L1 < 50%; 1 patient with unknown expression of PD-L1; 35 patients with stage III NSCLC
[16] Jiménez Galán R; September 2021	88	Spain, monocentric	23.0 months	7.9 months (95% CI: 1.2–14.6)	3.9 months (95% CI, 2.3–5.6)	/	2 patients with stage III B NSCLC; 7 patients with ECOG PS 3; 34.6% patients with ECOG PS ≥ 2
[17] Pons-Tostivint E; June 2023	141	France, multicentric	11.5 months	12-month survival rate: 66.1% (95% CI: 58–75.3)	10.6 months (95% CI 7.2—NR	/	/
[18] Izano MA, June 2023	341	USA: multicentric	10 months	18 months (95% CI: 14–22)	/	/	28 patients with PD-L1 < 50%, and 78 patients with unknown expression; 7 patients EGFR-mutated; 2 patients ALK-positive
[19] Descourt R, January 2023	845	France, multicentric	25.8 months	22.6 months (95% CI 18.5–27.4)	8.2 months (95% CI: 6.9- 9.5)	/	/
[20] Amrane K; April 2020	108	France, multicentric	8.2 months	15.2 months (95% CI, 13.9–NR)	10.1 months (95% CI: 8.8 to 11.4)	/	14 patients with stage III NSCLC
[21] Velcheti V; March 2022	566 (EHR cohort)	USA: multicentric	35.1 months	19.6 months (95% CI: 16.6–24.3)	/	/	All Patients had PS ECOG <2
	288 (spotlight coohrt)		38.4 months	21.1 (95% CI 16.2–28.9)	7.3 months (95% CI: 5.7–9.2)	/	
[22] Tamiya M; December 2019	213	Japan: Multicentric	11 months	17.8 months (95% CI: 17.8–NR)	8.3 months (95% CI: 6.0–10.7)	/	9 patients with ECOG PS 3 and 1 patients with ECOG PS 1; 6 patients EGFR mutated, 38 patients stage III.
[23] Goto Y, 5 August 2022	441	Japan: multicentric	13.5 months	12 and 24 months OS rate 72.2% (95% CI: 67.5–76.3) and 57.9% (95% CI: 50.8–64.3)	10 months (95% CI: 8.2–11.8)	rwTOT 5.6 (95% CI 4.4–6.7)	19% of patients with stage III NSCLC
[24] Mountzios G, March 2021	265	Italy, Spain, Greece, Switzerland: multicentric	/	22.5 months	/	TTP: 10.4 months	2 patients with ECOG PS 3
[25] Dudnik E; January 2021	203	Israel: multicentric	22.3 months	12.5 months (95% CI: 9.8–16.4)	/	TTD: 4.9 months (95% CI, 3.1–7.6)	9 patients with stage III NSCLC
[26] Tambo Y; September 2020	95	Japan: multicentric	8.8 months	12- and 24-month survival rate: 78.3% and 58.3%	6.1 months (95% CI: 3.64–8.56)	/	10 patients ECOG-PS 3–4; 29 patients with non metastatic NSCLC
[27] Cavaille F, February 2021	38	France: monocentric	7.6 months	11.08 months (95% CI: 5.98–NR)	6 months (95% CI 3–NR)	/	5 patients with ECOG PS 3; 2 patients with stage III NSCLC
[28] Frost N.; September 2021	153	Germany: multicentric	26.9 months	22.0 months (95% CI: 15.4–28.6)	8.2 months (95% CI 5.1–11.4)	/	6 patients with ECOG PS3; 29 patients with stage III NSCLC
[29] Cortellini A; November 2020	1026	Multicentric: Italy	14.6 months	17.2 months (95% CI: 15.3–22.3)	7.9 months (95% CI: 6.9–9.5)	/	/
[30] Velcheti V; November 2019	423	Multicentric: USA	18.4	18.9 months (95% CI 14.9–25.5)	6.8 months (95% CI 5.3–8.1)	/	/
	188		15.5	19.1 months (95% CI:12.6–NR)	/	/	15.4% of patients with non metastatic NSCLC

rwTOT: real-world time on treatment, TTP: time to progression.

## Data Availability

The raw data supporting the conclusions of this article will be made available by the authors without undue reservation.

## Supplementary Materials

The following supporting information can be downloaded at: https://www.mdpi.com/article/10.3390/cancers16101802/s1, Table S1: Reasons for pembrolizumab treatment discontinuation, Table S2: Response rate, Table S3: Adverse reaction management, Table S4: Univariable and multivariable model for PFS; Table S5: Univariable and multivariable model for OS; Figure S1: Kaplan–Meier curves of real-world PFS in subgroup of interest. (A) PD-L1 level (50–69%; 70–89%; ≥90%) (B) ECOG PS 0, 1 and 2 (C) Smoking status (former/current smoker vs. patient who never smoked); Figure S2: Kaplan–Meier curves of real-world OS in subgroup of interest. (A) Age (<75 years vs. ≥75 years). (B) Sex (male vs. female patients). (C) Histologic type (squamous NSCLC vs. non-squamous NSCLC). (D) Smoking status (former/current smoker vs. patient who never smoked). (E) ECOG PS 0, 1, and 2.
