# Supplementary material for: Real-World Safety and Outcome of First-Line Pembrolizumab Monotherapy for Metastatic NSCLC with PDL-1 Expression ≥ 50%: A National Italian Multicentric Cohort (“PEMBROREAL” Study)
Source: Cancers (Basel). 2024 May 8;16(10):1802. doi: 10.3390/cancers16101802 (PMC11119961; doi:10.3390/cancers16101802)

Supplementary Table S1: Reasons for Pembrolizumab Treatment discontinuation

Reason	Overall Patients n (%)
Progressive disease or clinical progressive disease	474 (53.9)
Unacceptable toxicity	55 (6.3)
Lost to follow up	39 (4.4)
Patient's decision	4 (0.5)
Clinical decision	46 (5.2)
Death	120 (13.6)
Not specified	1 (0.1)
Therapy is still ongoing at the cutoff data	141 (16.0)

Supplementary Table S2: Response Rate

Best overall response	Overall patients n (%)
Complete response	24 (2.7)
Partial response	155 (17.6)
Stable disease	204 (23.2)
Progressive disease	228 (25.9)
Not yet documented	269 (30.6)

Supplementary Table S3: Adverse reaction management

Action taken	Overall patients n (%)
Suspension of one or more drug	71 (8.07)
Permanent discontinuation of the treatment	47 (5.3)
Dose reduction of one or more drugs	0 (0.0)
Specific pharmacologic treatment for toxicity management	177 (20.1)
Hospitalization or prolongation of hospitalization	55 (6.25)

Supplementary Table S4: univariables and multivariable model for Rw-PFS

Variables	Univariable models			Multivariable model		
	Hazard Ratio	p-value	95% CI	Hazard Ratio	p-value	95% CI
Smoker/Former smoker vs no smoker	0.72	0.007	0.57-0.92	0.81	0.144	0.61-1.07
PDL1 70-89% vs PDL1 50-69%	0.90	0.368	0.73-1.11	0.82	0.125	0.64-1.05
PDL1 ≥90% vs PDL1 50-69%	0.64	0.001	0.49-0.83	0.62	0.001	0.46-0.83
PS ECOG 1 vs PS ECOG 0	1.36	<0.001	1.15-1.60	1.51	<0.001	1.20-1.89
PS ECOG 2 vs PS ECOG 0	2.56	<0.001	1.94-3.38	2.36	<0.001	1.50-3.69

Supplementary Table S5: univariables and multivariable model for Rw-OS

Variable by subgroup of therapy	Univariable models			Multivariable model		
	HR	p-value	95% CI	HR	p-value	95% CI
Age ≥75 years vs age <75	1.36	0.002	1.12-1.67	1.27	0.063	0.99-1.64
Female vs Male	0.77	0.015	0.62-0.95	0.83	0.188	0.64-1.09
No squamo vs squamo (main effect)	0.75	0.011	0.60-0.94	1.10	0.709	0.66-1.84
No squamo vs squamo (time dependent effect)	-	-	-	0.79	0.051	0.64-1.01
PS ECOG 1 vs PS ECOG 0 (main effect)	1.43	<0.001	1.18-1.74	3.09	<0.001	1.86-5.16
PS ECOG 2 vs PS ECOG 0 (main effect)	2.49	<0.001	1.82-3.40	5.89	<0.001	3.11-11.1
PS ECOG 1 vs PS ECOG 0 (time dependent effect)	-	-	-	0.67	<0.001	0.54-0.84
PS ECOG 2 vs PS ECOG 0 (time dependent effect)	-	-	-	0.53	<0.001	0.39-0.72
Smoker+ex smoker vs no smoker	1.37	0.045	1.01-1.87	1.32	0.077	0.97-1.82

Supplementary Figure S1: Kaplan – Meier curves for Rw-PFS in subgroup of interest. A) PD-L1 levels (50-69%; 70-89%; ≥ 90%) B) ECOG PS C) smoking status

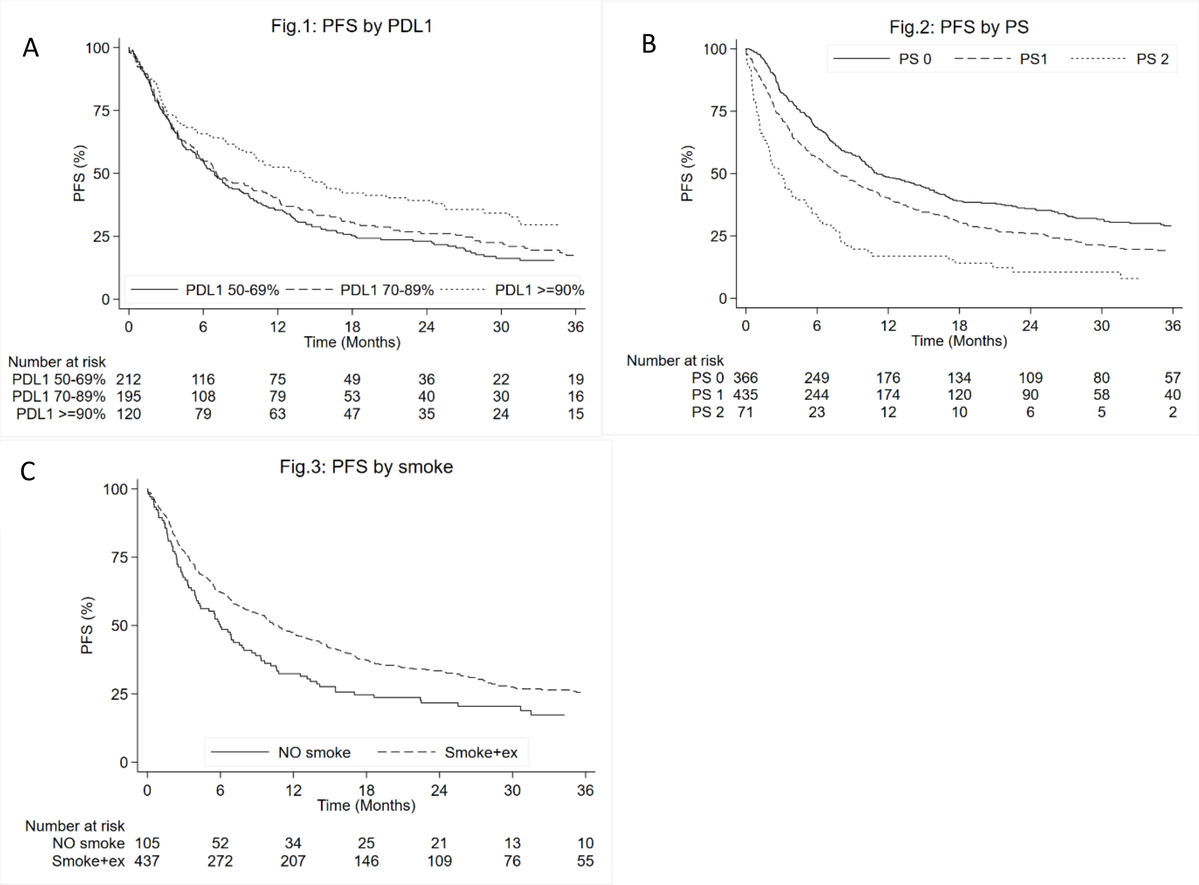

Supplementary Figure S2: Kaplan – Meier curves of real world OS in subgroup of interest. A) age (<75 years vs ≥ 75 years) . B) Gender C) histological type D) smoking status E) ECOG PS

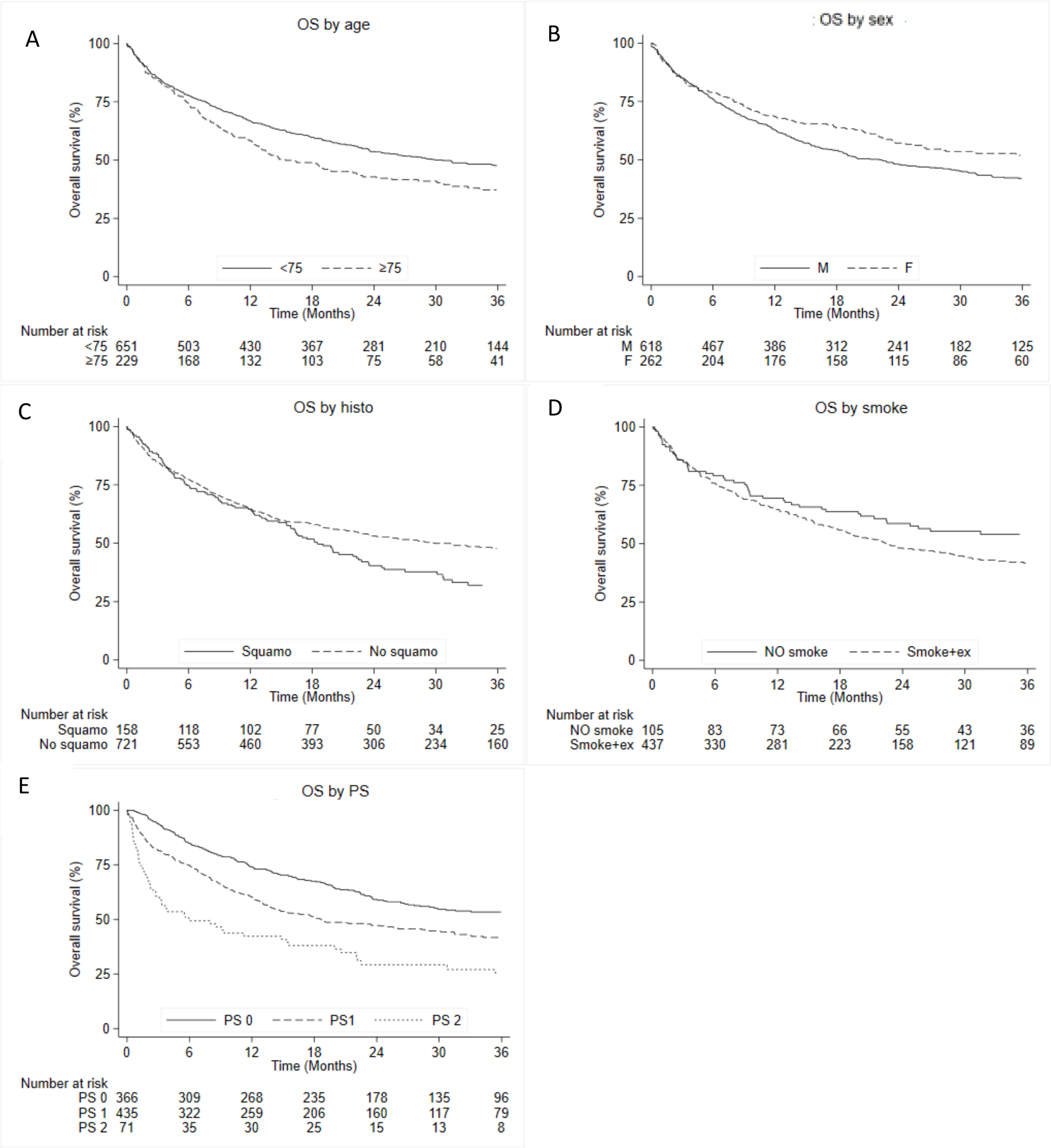

Supplement: Supplementary file 1 [file cancers-16-01802-s001.zip › cancers-2982780-supplementary.pdf]
